# Contemporary Management of Aortic Stenosis: Timing of Intervention and Lifetime Valve Management

**DOI:** 10.1111/eci.70245

**Published:** 2026-08-13

**Authors:** Ottavia Cozzi, Thomas Pilgrim

**Affiliations:** ^1^ Department of Cardiology, Bern University Hospital University of Bern Bern Switzerland

## Abstract

**Background:**

Calcific aortic stenosis (AS) is the most relevant valvular heart disease in aging populations and remains both underdiagnosed and undertreated. Screening studies reveal a high prevalence of unrecognized disease, while up to 40% of patients with symptomatic severe AS do not undergo valve intervention, resulting in poor survival. No pharmacologic therapy has yet altered disease progression, although targeted approaches such as lipoprotein(a) inhibition are under investigation.

**Methods:**

This paper provides a comprehensive perspective review of the evolving epidemiology, timing of intervention, and long‐term management of aortic stenosis, drawing on randomized trials and contemporary clinical data.

**Results:**

Randomized trials have challenged the traditional watchful waiting strategy in asymptomatic severe AS, showing that early aortic valve replacement (AVR) reduces cardiovascular events, though survival benefit depends on patient selection and timing. Moderate AS is increasingly recognized as a high‐risk condition, prompting ongoing trials evaluating earlier intervention. As transcatheter aortic valve implantation (TAVI) expands to younger, lower‐risk patients, long‐term issues—including conduction disturbances, paravalvular leak, cerebrovascular events, valve hemodynamics and durability, and redo feasibility—become central to care. Contemporary data suggest comparable mid‐term durability between TAVI and surgery, but lifetime management planning is essential.

**Conclusions:**

Optimal care of aortic stenosis requires earlier diagnosis, optimized procedural strategies, and a lifetime‐oriented approach to management, supported by continued investigation into pharmacologic therapy and the timing of intervention.

## Graphical abstract

As transcatheter treatment expands to younger and lower‐risk patients, aortic stenosis management is evolving from a single procedure to lifelong valve care. This review highlights the key challenges across the patient journey, from timely diagnosis and optimal intervention to durable valve performance, long‐term surveillance, and repeat intervention.

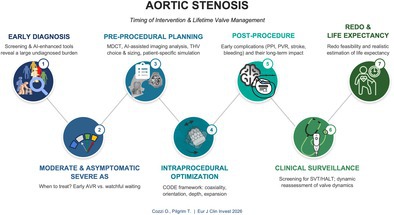

## 1 Epidemiology of Aortic Stenosis: Between Underdiagnosis and Undertreatment

Calcific aortic stenosis (AS) is the most prevalent valvular heart disease (VHD) in aging populations and, as such, represents an expanding public health challenge. In the OxVALVE study, systematic echocardiographic screening of asymptomatic adults aged > 65 years identified previously unrecognized, mostly mild, VHD in 51% of the participants; 34% of them had aortic sclerosis, the pathological precursor of AS. Overt AS was detected in 1.3% of the participants [1]. More recently, the PREVUE‐VALVE study provided contemporary, nationally representative U.S. data demonstrating that moderate or greater VHD affects 8.2% of adults aged 65–85 years, while AS is present in 3.1% of the screened population and its prevalence rises steeply with age [2]. Despite increasing awareness, the true burden of AS remains underestimated, as diagnosis continues to rely on access to echocardiography and experienced interpretation, and many patients remain asymptomatic or labelled as such until advanced stages.

Beyond underdiagnosis, undertreatment remains a major gap in care. Up to 40% of elderly patients with symptomatic severe AS do not undergo valve intervention [3], and fewer than two‐thirds of those with echocardiographically confirmed severe disease receive aortic valve replacement (AVR) within 4 years. As a result, many patients remain exposed to the markedly elevated mortality associated with untreated AS [4, 5].

These data frame AS as a condition in which outcomes are powerfully shaped by the timing of diagnosis, referral and definitive intervention.

## 2 Disease Modifying Therapies for AS

Despite decades of investigation, no pharmacologic therapy has convincingly altered the natural history of calcific AS (Table 1). Consequently, patients with early disease—ranging from aortic sclerosis to moderate AS—often experience prolonged periods of ‘watchful waiting’, during which progressive valvular and myocardial damage may occur. Early strategies focusing on intensive lipid lowering with statins or statin–ezetimibe combinations failed to slow disease progression, underscoring that advanced calcific AS is largely unresponsive to nonspecific lipid modification [6, 7, 8, 17]. Similarly, other strategies aimed at modulating valvular calcification, such as vitamin K2 and D supplementation, have not shown convincing effects on the progression of aortic valve calcification [10].

**TABLE 1:** Pharmacologic therapies investigated for calcific aortic stenosis: Mechanisms, key clinical trials, and current evidence.

Therapeutic strategy	Mechanistic target	Key trial(s)	Population	Main findings/status
**Therapies targeting valve disease initiation and calcification**				
Statins	LDL‐C lowering; anti‐inflammatory effects	SALTIRE [6], ASTRONOMER [7]	Mild–moderate AS or asymptomatic AS	No reduction in AS progression, valve calcification, or clinical outcomes
Statin + ezetimibe	Intensive lipid‐lowering therapy	SEAS [8]	Asymptomatic mild–moderate AS	No effect on AS progression
PCSK9 inhibitors	Reduction of LDL‐C and lipoprotein(a)	FOURIER exploratory analysis [9]	Patients with cardiovascular disease	Lower incidence of AS events observed; hypothesis‐generating evidence for role of Lp(a)
Lipoprotein(a)–lowering therapies (pelacarsen)	Targeting Lp(a) and oxidized phospholipid pathways	Lp(a)HORIZON, Lp(a)FRONTIERS‐CAVAS (all ongoing)	Patients with elevated Lp(a) and early AS	Ongoing trials evaluating whether Lp(a) lowering slows AS progression
Vitamin K (menaquinone)	Activation of matrix Gla protein; inhibition of vascular calcification	AVADEC [10], BASIK‐2 (ongoing)	Aortic valve calcification or mild–moderate AS	First randomized trial largely neutral for calcification progression. Further evidence awaited from ongoing trial
Bisphosphonates/anti‐resorptive therapy	Inhibition of hydroxyapatite crystal formation and osteoclast activity	SALTIRE II [11]	Calcific AS	No significant effect on AV calcification progression
**Therapies targeting valvular calcification signalling pathways**				
sGC activators (e.g., ataciguat)	Restoration of nitric oxide–independent soluble guanylate cyclase signalling	A Study Evaluating the Effects of Ataciguat on Aortic Valve Calcification [12]; KATALYST‐AV (ongoing)	Moderate calcific AS	Phase II trial showed slower progression of valve calcification with signals of improved valvular and ventricular function. Phase III studies ongoing
DPP‐4 inhibition (evogliptin)	Endothelial and metabolic pathways	DIP‐CAVD [13], EVOID‐AS (ongoing)	Mild–moderate AS	No significant effect on progression of AV calcification. Exploratory PET imaging suggested reduced microcalcification activity. Further evidence awaited from ongoing trial
Anti‐inflammatory therapy (colchicine)	Inhibition of inflammatory signalling pathways	CHIANTI, COPAS (both ongoing)	Moderate AS	Ongoing trials evaluating effects on valve calcification progression
PPAR‐γ modulation (pioglitazone)	Metabolic and inflammatory pathway modulation	Pioglitazone in Calcific AVD trial (ongoing)	Mild–moderate AS	Ongoing trial assessing mortality and disease progression
**Therapies targeting myocardial consequences of AS**				
Renin–angiotensin system blockade (Ramipril/ARB)	Modulation of myocardial remodelling and fibrosis	RIAS [14], RASTAVI [15], ARBAS (ongoing)	Mild–moderate AS; after AVR	Improved LV remodelling and reduced HF readmissions after TAVI with ACE‐i; no clear effect on valve progression or major outcomes. Ongoing trial to evaluate potential effects of ARB on LV remodelling
SGLT2 inhibitors (Dapagliflozin, ENAVOgliflozin)	Hemodynamic unloading and HF benefit	DAPA‐TAVI [16], ENAVO‐TAVR (ongoing)	Severe AS undergoing TAVI with prior HF	Reduced composite of death or HF worsening; effect likely myocardial rather than valve‐modifying. Further evidence awaited from ongoing trial

*Note:* Summary of completed and ongoing clinical studies evaluating pharmacologic strategies targeting lipid metabolism, inflammation, calcification pathways, endothelial dysfunction, and myocardial remodelling in patients with calcific aortic stenosis.

Abbreviations: ACE‐i, angiotensin‐converting enzyme inhibitor; ARB, angiotensin receptor blocker; AS, aortic stenosis; AV, aortic valve; AVR, aortic valve replacement; DPP‐4, dipeptidyl peptidase‐4; HF, heart failure; LDL‐C, low‐density lipoprotein cholesterol; Lp(a), lipoprotein(a); PET, positron emission tomography; PPAR‐γ, peroxisome proliferator–activated receptor gamma; RAAS, renin–angiotensin–aldosterone system; sGC, soluble guanylate cyclase; TAVI, transcatheter aortic valve implantation.

Observational data from patients treated with PCSK9 inhibitors [9] and genome‐wide association studies [18] have identified apo B‐containing lipoproteins—particularly lipoprotein(a)—as causal contributors to early aortic valve calcification, renewing interest in targeted lipid‐lowering approaches [19, 20]. Ongoing randomized trials with pelacarsen, an antisense oligonucleotide targeting apolipoprotein(a), including Lp(a)HORIZON (NCT04023552) and Lp(a)FRONTIERS CAVAS (NCT05646381), will determine whether modifying this pathway can alter cardiovascular outcomes and AS progression. In parallel, modulation of oxidative stress and soluble guanylate cyclase signalling, exemplified by ataciguat, represents another emerging strategy [12]. Although these approaches remain investigational, they highlight a paradigm shift toward earlier, biology‐driven intervention potentially relevant before irreversible myocardial and valvular damage occur.

Although these valve‐targeted strategies remain investigational, the myocardium represents a parallel and increasingly relevant therapeutic target. In patients with concomitant AS and heart failure (HF), optimization of guideline‐directed medical therapy is often limited by intolerance related to fixed afterload and hemodynamic instability, requiring cautious titration of agents affecting blood pressure or preload. Renin–angiotensin system (RAS) blockade has been the most extensively studied approach: experimental and observational data link angiotensin II to valvular inflammation, left ventricular hypertrophy, and fibrosis [14], and RAS inhibition after AVR has been associated with reduced HF readmissions and greater regression of left ventricular hypertrophy [15, 21]. More recently, sodium–glucose cotransporter 2 inhibitors demonstrated a 28% relative reduction in the composite of death or worsening HF in patients undergoing TAVI with a prior HF event, an effect likely mediated through myocardial rather than valve‐level mechanisms, and a signal that cardiometabolic optimization may meaningfully complement procedural care in this population [16].

## 3 Timing of Intervention and Early TAVI

### 3.1 Asymptomatic Severe Aortic Stenosis

The optimal timing of AVR in asymptomatic severe AS is among the most debated issues in contemporary VHD management. Observational data historically suggested a relatively favourable short‐term prognosis under surveillance, with annual mortality rates of approximately 1%–2% and symptom development occurring in most patients within 1–2 years of diagnosis [22]. This perceived short‐term safety formed the basis of the traditional ‘watchful waiting’ strategy.

However, accumulating evidence indicates that prolonged exposure to severe valvular obstruction promotes maladaptive myocardial remodelling and irreversible cardiac damage, even before symptom onset, with important long‐term prognostic consequences [23, 24, 25, 26]. These insights have prompted re‐evaluation of whether delaying intervention until symptom development may allow irreversible myocardial damage to occur.

Support for early intervention first emerged from surgical trials. The RECOVERY and AVATAR trials demonstrated that early surgical AVR (SAVR) reduced mortality and HF hospitalization compared with conservative management in younger patients with severe or very severe AS [27, 28]. More recently, two randomized trials, EVoLVeD and EARLY TAVR, extended this question to transcatheter aortic valve implantation (TAVI) [29, 30].

EVoLVeD randomized patients with asymptomatic severe AS and midwall myocardial fibrosis on cardiac magnetic resonance to early intervention (SAVR or TAVI) versus clinical surveillance. Although early intervention did not reduce the composite endpoint of all‐cause death or unplanned AS hospitalization, it was associated with fewer NYHA class II–IV symptoms at 12 months, and a reduction in aortic‐stenosis related hospitalizations. In contrast, the EARLY‐TAVR trial demonstrated that early TAVI in patients with severe asymptomatic AS significantly reduced the composite of death, stroke, or unplanned cardiovascular hospitalization compared with surveillance at a median follow‐up of 3.8 years [29]. The difference was driven by lower rates of unplanned hospitalizations for cardiovascular causes, while rates of mortality were comparable between patients with early interventions or active surveillance.

A meta‐analysis integrating these trials showed that early AVR significantly reduced unplanned cardiovascular or HF hospitalization (14.6% vs. 31.9%; HR 0.40) and stroke (4.5% vs. 7.2%; HR 0.62), but not all‐cause or cardiovascular mortality at a mean follow‐up of 4.1 years [31]. Differences in patient age, baseline risk, and the duration of exposure to symptomatic severe AS in surveillance arms may partly explain the different findings in the trials, as shown in Table 2. Notably, crossover rates were high across trials (> 70% in all but AVATAR), highlighting that watchful waiting frequently results in delayed rather than avoided intervention.

**TABLE 2:** Randomized control trials evaluating early intervention in aortic stenosis.

Study name	Patient population	Treatment/randomization	Primary endpoints and results	Main results		Timing and modality of surveillance	Crossover rate and reason
Asymptomatic severe AS							
RECOVERY trial (NCT01161732)*	Asymptomatic very severe AS^a^ *Mean age: 64.5 years* *Euroscore II: 0.9%* *Bicuspid AV: 61%*	SAVR vs. conventional treatment *N* = 145 Median time to SAVR in the intervention group: 23 days	Operative mortality or cardiovascular mortality at 4 years	SAVR 1% vs. surveillance 15%; HR 0.09, 95% CI 0.01–0.67, *p* = 0.003		Annual echocardiography, clinical FU at 3,6,12 months and then every 6 months	Crossover: 72.6%, median 700 days Reason: 43 pts. symptoms; 9 admitted for AVS
AVATAR (NCT02436655)*	Asymptomatic severe AS STS < 8% *Mean age: 67 years* *STS‐PROM: 1.7%* *Bicuspid AV: 14%*	SAVR vs. conservative treatment *N* = 157 Median time to SAVR in the intervention group: 55 days	Composite: all‐cause death or MACE (3 years)	SAVR 15.2% vs surveillance 34.7%; HR: 0.46; 95% CI: 0.23–0.90; *p* = 0.021		Not specified	Crossover: 31.6%, median 476 days Reason: 60% symptoms, 16% AS progression, 4% decreased LVEF, 20% combined
EVoLVeD (NCT03094143)*	Asymptomatic severe AS CMR‐detected midwall fibrosis *Mean age: 73.4 years* *STS‐PROM risk: not reported* *Bicuspid AV: 29%*	AVR (TAVI 25% pts/SAVR 75% pts.; any CE mark–approved) vs. clinical surveillance *N* = 224 *(planned 356 pts)* Median time to AVR in the intervention group: 152 days	Composite: all‐cause death or AS‐related hospitalization (42 months)	AVR 18% vs surveillance 23%; HR: 0.79; 95% CI: 0.44–1.43; *p* = 0.44		According to local policy. Follow‐up in the study with annual telephonic contact	Crossover: 77%, median 614 days Reason: symptom 72%, 15% urgent inpatient surgery
EARLY TAVR (NCT03042104)*	Asymptomatic severe AS Age ≥ 65 years & anatomy suitable for TAVI with BEV *Mean age: 75.8 years* *STS‐PROM risk: 1.8%* *Bicuspid AV: 8.4%*	TAVI (SAPIEN 3/3 Ultra) vs. clinical surveillance *N* = 901 Median time to TAVI in the intervention group: 14 days	Composite: all‐cause death, stroke, unplanned CV hospitalization (2 years)	TAVI 26.8% vs surveillance 45.3%; HR: 0.50; 95% CI: 0.40–0.63; *p* < 0.001		Follow‐up in the study at 1, 2, 3, 5 and 10 years	Crossover: 87%, median 333 days Reason: symptom 97.2%, other causes 2.8% (↓ LVEF, ↑ 3xNT‐proBNP, treatment of non‐cardiac disease, Peak vel > 5 m/s)
DANAVR (NCT03972644)^†^	Asymptomatic severe AS And ≥ 1 at‐risk feature E/e′ > 13 LAVi > 34 mL/m^2^ NT‐proBNP ≥ 3× normal Abnormal GLS	Early AVR (TAVI or SAVR) Planned *N* = 1700	All‐cause death (at 5 years; 379 events)	Ongoing	Recruiting	Not specified	Ongoing
EASY AS (NCT04204915)^†^	Asymptomatic severe AS	Early AVR (within 3 months, TAVI or SAVR) vs. conservative therapy Planned *N* = 2844	Composite: all‐cause death or Hf hospitalization (at minimum 3 years)	Ongoing	Recruiting	Clinical assessment every 6 months up to 36 months; echo every 6 months	Ongoing
Moderate AS							
TAVR UNLOAD (NCT02661451)*	Moderate AS NYHA II and EF < 50% *Mean age: 77 years* *Mean STS risk: 4.4%*	TAVI (SAPIEN 3/3 Ultra) vs. clinical surveillance *N* = 178 *(Planned 600 pts)* Median time to AVR in the intervention group: 13 days	Composite: all‐cause death, disabling stroke, AS or HF hospitalization, change in KCCQ from baseline (2 years, hierarchical)	TAVI 47.6% wins vs. surveillance 36.6% wins (WR: 1.31; 95% CI: 0.91–1.88; *p* = 0.14) Change in KCCQ at 1 year: 12.8 ± 21.9 TAVI vs. 3.2 ± 22.8 in surveillance, *p* = 0.018		Follow‐up visits at 1,6, 12 months, 2 years	Crossover: 43%, median 360 days
PROGRESS (NCT04889872)^†^	Age ≥ 65 years Moderate AS Symptoms of AS (angina or dyspnea), or signs of cardiac damage/dysfunction	TAVI (SAPIEN 3/3 Ultra/3 Ultra Resilia) vs. clinical surveillance Planned *N* = 2250	Composite: all‐cause death and HF hospitalization or events (2 years)	Ongoing	Recruiting	Not specified	Not specified
EXPAND TAVR II (NCT05149755)^†^	Age ≥ 65 years Moderate AS And ≥ 1 at‐risk feature: Symptoms of AS (NHYA II, ↓ functional capacity) HF event or hospitalization within the last year NT‐pro‐BNP ≥ 600 pg/mL Persistent or paroxysmal AF Systolic/diastolic dysfunction at echo^b^ Elevated aortic valve calcium (> 1200 AU women, > 2000 AU men)	TAVI (Evolut PRO+, or Evolut FX, & GDMT) vs. GDMT *N* = 650 pts	Composite: all‐cause death and HF events, AV re‐interventions (2 years)	Ongoing	Enrollment completed	Not specified	Not specified

*Note:* ‘*’ indicates completed trials, ‘†’ indicates ongoing ones.

Abbreviations: AS, aortic stenosis; AU, Agatston Units; AV, aortic valve; AVR, aortic valve replacement; AVS, acute valve syndrome; BEV, balloon expandable valves; CMR, cardiac magnetic resonance; FU, follow‐up; GDMT, Guideline Directed medical Therapy; GLS, global longitudinal strain; HF, heart failure; HR, hazard ratio; IQR, interquartile range; KCCQ, Kansas City Cardiomyopathy Questionnaire; LAVi, left atrial volume indexed; LV, left ventricle; LVEF, left ventricular ejection fraction; NT‐proBNP, *N*‐terminal pro–B‐type natriuretic peptide; NYHA, New York Heart Association; pts., patients; SAVR, surgical aortic valve replacement; STS, Society of Thoracic Surgeons Predicted Risk of Mortality score; TAVI, transcatheter aortic valve implantation.

^a^ Very severe AS defined as: a critical stenosis in the AV area ≤ 0.75 cm^2^ fulfilling one of the following criteria: a peak aortic velocity ≥ 4.5 m/s or a mean transaortic pressure gradient ≥ 50 mmHg on Doppler echocardiography.

^b^ E/e′ ≥ 14, or GLS ≤ 16%, or diastolic dysfunction ≥ grade 2, or LVEF < 60%, or stroke volume index < 35 mL/m^2^.

**FIGURE 1:**
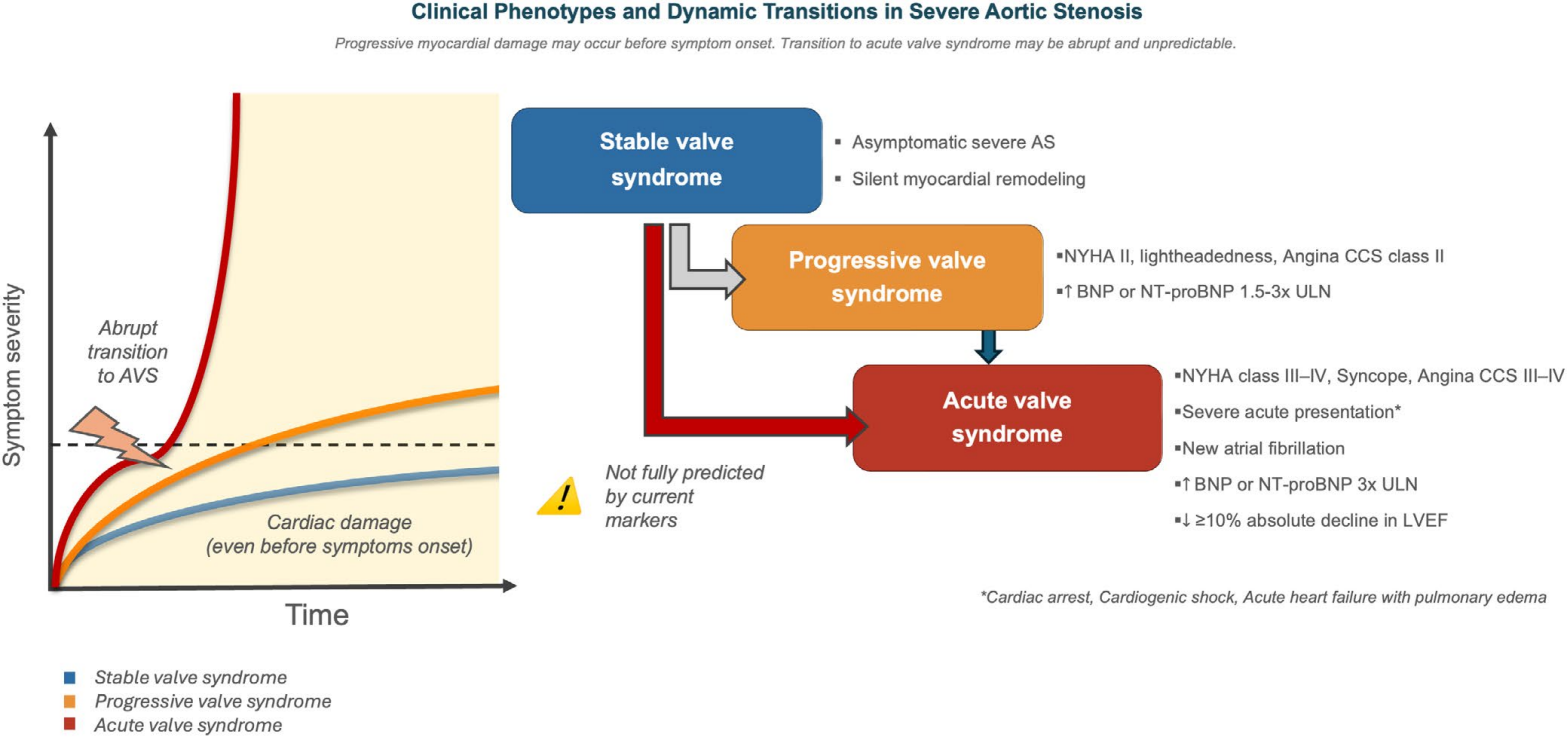
Clinical phenotypes and dynamic transitions in severe aortic stenosis (AS). Progressive myocardial damage may occur before overt symptom onset. Although many patients transition gradually from stable to progressive disease, some may experience an abrupt deterioration to acute valve syndrome (AVS), not fully predicted by current markers. Three phenotypes are proposed: Stable valve syndrome, progressive valve syndrome, and acute valve syndrome. AS, aortic stenosis; BNP, B‐type natriuretic peptide; CCS, Canadian Cardiovascular Society; LVEF, left ventricular ejection fraction; NT‐proBNP, *N*‐terminal pro–B‐type natriuretic peptide; NYHA, New York Heart Association; ULN, upper limit of normality.

In the EARLY‐TAVR, approximately 40% of patients undergoing delayed AVR transitioned from an asymptomatic state directly to acute and severe clinical presentations, challenging the traditional concept of gradual symptom progression. To better characterize these clinical patterns, a new classification has been proposed, distinguishing: (1) stable valve syndrome (absence of AS‐related signs or symptoms), (2) progressive valve syndrome (PVS; NYHA class II symptoms or NT‐proBNP ≥ 1.5 and < 3× the age‐adjusted upper limit of normal) and (3) acute valve syndrome (AVS), defined by NYHA class III–IV symptoms, syncope, new‐onset atrial fibrillation, ventricular arrhythmia, resuscitated cardiac arrest, HF hospitalization or pulmonary edema, NT‐proBNP ≥ 3× upper limit of normal, or a > 10% decline in left ventricular ejection fraction [32] (Figure 1).

In a subanalysis of the EARLY‐TAVR trial, patients presenting with AVS who underwent delayed AVR had more than a twofold higher risk of death, stroke, or HF hospitalization compared with those treated with early TAVI (14.9% vs. 6.8%; HR: 2.37) [33] and had less cardiac damage recovery after TAVI. In contrast, outcomes in the PVS subgroup were similar between early and delayed intervention. Consistently, real‐world data from a multicenter cohort of 17,838 patients show that a substantial proportion present directly with AVS despite close follow‐up, and that these patients experience worse outcomes after AVR compared with those in stable or progressive stage [34, 35]. In addition, in the prospective AS DEFER study, deferral of AVR in patients with severe aortic stenosis was associated with an increased risk of hospitalization for valve‐related symptoms or worsening heart failure [36]. Collectively, these data suggest that once severe AS is established, patients enter a vulnerable phase characterized by silent myocardial injury, unpredictable progression, and risk of abrupt decompensation [37]. In such settings, watchful waiting may represent deferred harm rather than prudent conservatism, particularly when surveillance intensity and rapid access to AVR differ from those achieved in clinical trials.

Identifying patients at risk for rapid deterioration remains a critical unmet need. Predictors of AVS include inability to exercise, diabetes, elevated NT‐proBNP, and increased left atrial volume at baseline [38], whereas midwall fibrosis alone may not adequately guide timing of intervention [30]. Emerging strategies, including machine learning–based predictive models [39], and remote patient monitoring with wearable technologies [40, 41, 42], may enhance early detection of clinical instability and refine individualized decision‐making. A complementary, still investigational axis is molecular fingerprinting. Multi‐omic profiling, spanning the (epi)genome, transcriptome, proteome, and metabolome of blood and valvular tissue, is increasingly proposed as a route to a holistic molecular fingerprint of calcific aortic valve disease, capable of identifying circulating biomarkers and disease‐driving pathways that could refine prediction models of progression beyond clinical and echocardiographic staging alone [43]. In practice, this is already emerging in smaller‐scale form: large‐scale plasma proteomic profiling has identified circulating protein signatures associated with incident AS‐related hospitalization [44], and a small panel of circulating microRNAs, combined with echocardiographic parameters into a multiparametric model, improved diagnostic and risk‐stratification accuracy over either modality alone [45]. Integrating such molecular fingerprints with longitudinal clinical evaluation and imaging‐based severity indices may ultimately offer a more individualized answer to the question of ideal timing of intervention in aortic stenosis.

Ongoing trials, including EASY‐AS (NCT04204915) [46], DANAVR (NCT03972644) are expected to further clarify the role of earlier intervention in asymptomatic severe AS (Table 2).

### 3.2 Moderate AS

Population‐based analyses demonstrate that moderate AS is associated with more than a twofold increase in all‐cause mortality compared with age‐ and sex‐matched controls. Adverse cardiac remodelling may already be present at this stage and may not be fully reversible [47]. Moreover, observational data indicate that patients with moderate AS and advanced stages of cardiac damage have a higher 10‐year mortality compared to patients with severe AS and early stages of cardiac damage [48].

The progression from moderate asymptomatic to severe symptomatic AS is highly heterogeneous. A meta‐analysis reported average annual progression rates of +4.10 mmHg in mean transvalvular gradient, −0.08 cm^2^ in aortic valve area, and +158.5 Agatston Units in aortic valve calcium score, with increasing baseline AS severity predicting more rapid progression [49]. Contemporary registries report AVR rates ranging from 45.5% at 5 years in moderate AS [3] to 36.7% of patients with moderate‐to‐severe AS within four years [5]. Emerging data from machine learning–based models trained on longitudinal echocardiographic and clinical datasets have demonstrated strong predictive performance in identifying patients with mild‐to‐moderate AS who are likely to progress to severe disease within 2–5 years, outperforming conventional clinical predictors [50].

Currently U.S. and European guidelines recommend AVR in moderate AS only when patients are undergoing cardiac surgery for another indication [51]. However, given the improving safety profile of contemporary AVR and the adverse prognosis associated with untreated moderate AS, expanding the therapeutic window to include selected patients with moderate disease has become an area of active investigation. Observational data showed, that AVR was associated with a lower mortality in patients with moderate AS and advanced cardiac damage compared to conservative care [48], The TAVR UNLOAD randomized trial addressed this question in patients with moderate AS and HF with reduced ejection fraction [52]. Although TAVI was safe and resulted in early improvements in quality of life, it did not significantly reduce the composite hierarchical endpoint of death, disabling stroke, disease‐related hospitalizations, and quality of life status at longest follow‐up. Several factors may have influenced these findings, including advanced baseline myocardial disease, crossover to TAVI in the surveillance arm, absence of fully contemporary HF therapies, and reduced statistical power. Nonetheless, the results emphasize the critical importance of patient selection and of distinguishing afterload‐driven ventricular dysfunction from intrinsic cardiomyopathy. Ongoing randomized trials aim to further refine this question. PROGRESS (NCT04889872) and Evolut EXPAND II (NCT05149755) are evaluating TAVI in patients with moderate AS and preserved ejection fraction, incorporating clinical symptoms and objective markers of cardiac damage such as diastolic dysfunction, elevated biomarkers and atrial fibrillation. Together, these studies are expected to refine risk stratification and clarify whether earlier AVR in carefully selected patients with moderate AS can favourably alter the natural history of the disease (Table 2).

## 4 Long‐Term Issues After TAVI in Patients With Longer Life Expectancy

As TAVI is being performed earlier in the disease trajectory and expanded to younger patients with a longer life expectancy, long‐term device‐ and procedure‐related sequelae become increasingly relevant. Although suboptimal implantation may be driven by anatomical complexity, growing awareness of the factors influencing valve durability and reintervention feasibility underscores the importance of achieving optimal results at the index procedure. Of note, although the term transcatheter aortic valve replacement (TAVR) is commonly used in the literature as equivalent to TAVI, it is something of a misnomer: unlike SAVR, in which the diseased leaflets are excised and a new prosthesis is sutured into the annulus, TAVI does not remove the native valve but rather compresses and pins the calcified leaflets against the aortic wall, upon which the bioprosthesis is deployed. This distinction is not merely semantic, as retained native leaflet and annular calcific material underlie several of the device–host interactions, including conduction disturbances and paravalvular regurgitation, discussed below.

The following sections summarize the main long‐term considerations after TAVI and discuss emerging strategies to optimize the first implant and preserve future treatment options within a lifetime management framework.

### 4.1 Conduction Disturbances and New Permanent Pacemaker

**FIGURE 2:**
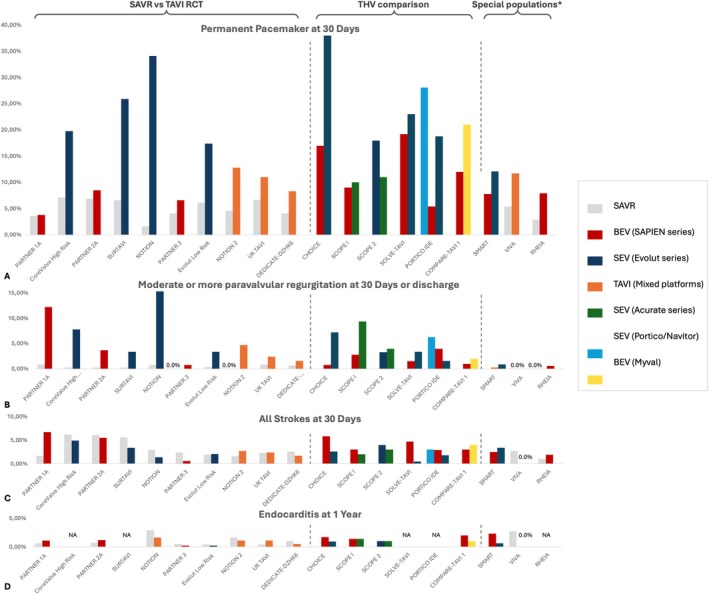
Procedural and valve‐related complications across pivotal randomized trials. Incidence of key procedural and valve‐related complications reported across pivotal randomized controlled trials comparing transcatheter aortic valve implantation (TAVI) with surgical aortic valve replacement (SAVR), or comparing different transcatheter heart valve (THV) platforms head‐to‐head. (A) New permanent pacemaker implantation at 30 days. (B) Moderate or greater paravalvular regurgitation at 30 days or hospital discharge. (C) All strokes at 30 days. (D) Infective endocarditis at 1 year. Bars are colour‐coded by treatment arm/THV platform. Trials are grouped along the x‐axis into three categories, separated by dashed vertical lines: TAVI‐vs‐SAVR randomized trials, THV‐vs‐THV (platform) randomized trials, and trials restricted to a special population. *Special‐population trials are grouped together by the population they enrolled (small aortic annuli or female sex) rather than by comparison design, and so include both a TAVI‐vs‐THV platform comparison (SMART) and TAVI‐vs‐SAVR comparisons (VIVA, RHEIA). ‘0.0%’ indicates a reported event rate of zero; ‘NA’ indicates the endpoint was not reported for that trial at the specified time point. Data are derived from each trial's primary publication. BEV, balloon‐expandable valve; CHOICE, comparison of balloon‐expandable vs. self‐expandable valves in patients undergoing transcatheter aortic valve replacement trial; COMPARE‐TAVI 1, SAPIEN 3 versus myval transcatheter heart valves for transcatheter aortic valve implantation trial; CoreValve High Risk, CoreValve US pivotal high risk study; DEDICATE‐DZHK6, German centre for cardiovascular research trial 6; NOTION, NOrdic aortic valve intervention trial; PARTNER, placement of AoRTic TraNscathetER valves trial; PORTICO‐IDE, portico investigational device exemption trial; RHEIA, Randomized reseArch in womEn All‐comers with aortic stenosis trial; SAVR, surgical aortic valve replacement; SCOPE, safety and efficacy comparison of two TAVI systems; SEV, self‐expanding valve; SMART, SMall Annuli Randomized To Evolut or SAPIEN trial; SOLVE‐TAVI, comparison of second‐generation self‐expandable versus balloon‐expandable valves and general versus local anaesthesia in transcatheter aortic valve implantation trial; SURTAVI, surgical replacement and transcatheter aortic valve implantation trial; TAVI, transcatheter aortic valve implantation; THV, transcatheter heart valve; UK TAVI, United Kingdom transcatheter aortic valve implantation trial; VIVA, transcatheter aortic valve replacement versus surgical aortic valve replacement for treating elderly patients with severe aortic stenosis and small aortic annuli trial.

Cardiac conduction disturbances (CCD) and new permanent pacemaker implantation (PPI) are still frequent after TAVI (Figure 2). Data from the TVT registry shows decreased incidence of new left bundle branch block (LBBB) after TAVI from nearly 20% in 2016 to 14% in 2022 [53] and a median rate of PPI implantation of 9.7% in 2020, with large variability across centers, secondary to transcatheter heart valve (THV) platforms used and non‐consistent protocols for post‐procedural CCD management [54]. Both new LBBB and PPI have been associated with adverse long‐term outcomes [55, 56, 57], as chronic ventricular pacing may promote pacing‐induced cardiomyopathy [58, 59] and worsen tricuspid regurgitation. Importantly, many patients receiving a pacemaker after TAVI are not pacemaker‐dependent at follow‐up, underscoring the need for individualized risk stratification and avoidance of unnecessary PPI [60]. Procedural strategies to reduce CCD include high implantation techniques (the cusp‐overlap technique for self‐expanding valves [61, 62], SEV, or high‐implantation technique for the SAPIEN valve [63]), implantation techniques tailored to the length of the membranous septum (MInimizing Depth According to the Septum, MIDAS [64], approach), and avoidance of excessive THV oversizing while tailoring balloon pre‐and post‐dilatation. This should be balanced against a potentially higher risk of coronary obstruction in re‐do TAVI with a high implant. Newer‐generation THVs incorporate design features such as radiopaque markers, larger cells at the level of the coronary ostia, and enhanced delivery control, facilitating more precise positioning and reducing septal injury [65, 66].

Beyond mechanical injury, inflammation may represent a complementary mechanism contributing to CCD. Systemically, TAVI induces an inflammatory response syndrome in 22%–63% of transfemoral procedures [67]. Locally, periannular edema may transiently compress the conduction system, leading to atrioventricular block and increasing the likelihood of PPI [68, 69]. Two randomized trials explored whether anti‐inflammatory therapy could reduce CCD after TAVI. In the Co‐STAR trial, periprocedural colchicine reduced new‐onset atrial fibrillation or CCD requiring PPI at 30 days (10% vs. 25%) [70]. Similarly, in the GLUCO‐TAVI trial, perioperative corticosteroids showed a numerically lower PPI rate without statistical significance [71]. Whether anti‐inflammatory modulation translates into meaningful clinical benefit remains uncertain and warrants larger biomarker‐guided randomized trials [72].

Following TAVI, structured management algorithms [73], ambulatory electrocardiographic monitoring [74, 75, 76, 77] or electrophysiological studies [78, 79] may enable early detection of delayed high‐grade block while avoiding unnecessary PPI. Finally, when pacing is unavoidable, physiologic approaches, such as His‐bundle or left bundle branch area pacing, might better preserve ventricular synchrony than conventional right ventricular pacing [80, 81]. These novel approaches are under active investigation in randomized trials and may redefine post‐TAVI rhythm management in the upcoming years [82].

### 4.2 Paravalvular Regurgitation

Paravalvular regurgitation (PVR) has improved substantially with contemporary valve design and CT‐based sizing (Figure 2), yet mild PVR remains common, with low‐risk trials reporting rates of 29% in balloon expandable valves (BEVs) and 36% in SEVs [83, 84]. Newer THV generations are associated with even lower risk of mild PVR thanks to improved sealing skirts and radial force [85, 86]. Whether mild PVR is associated with poorer clinical impact after TAVI appears inconsistent across studies, likely due to different patient populations (mild PVR is less well tolerated in higher risk patients [87, 88] and patients with no prior aortic regurgitation [89]), variable follow‐up [88, 90, 91], poor reproducibility of echocardiographic assessment of PVR [92], inconsistent stratification between mild‐to‐moderate and mild PVR across different studies [90]. Current prevention of PVR relies on tailored procedural planning for valve and balloon sizing, assessment of annular [93] and LVOT calcification [94], as well as improved THV technology. Intraprocedurally, a multiparametric PVR assessment is advisable, including invasive hemodynamics [95, 96]. Finally, redo TAVI or PVR closure with a plug is feasible in case of residual significant PVR [97].

### 4.3 Post‐Procedural Hemodynamics and Small Annuli

Whether post‐procedural hemodynamics and prosthesis–patient mismatch (PPM) meaningfully influence long‐term outcomes after TAVI remains debated. Recently, great attention has been focused on patients with small aortic annuli, commonly defined as an annular area ≤ 430 mm^2^ on contrast‐enhanced CT, a phenotype frequently encountered in women undergoing TAVI [98, 99]. Use of smaller prosthesis platforms in this population is associated with less favourable post‐procedural hemodynamics and higher residual gradients, particularly with BEVs [85, 98, 99, 100, 101]. In the SMART trial [102], moderate or severe PPM occurred in 35.3% of patients with small annuli treated with BEVs compared with 11.2% receiving SEVs, with corresponding bioprosthetic valve dysfunction (BVD) rates of 41.6% versus 9.4% at 12 months, findings largely driven by residual gradients > 20 mmHg and severe PPM. Although multiple contemporary studies indicate that residual gradients do not translate into adverse long‐term clinical events after TAVI [85, 98, 100, 101, 102, 103, 104, 105], recent evidence suggests a stepwise increase of structural valve deterioration rates with increasing early residual mean postprocedural gradient up to 10 years follow‐up in patients undergoing TAVI with SEVs [106]. Whether higher gradients have greater relevance for symptoms, ventricular remodelling, and the feasibility of future redo procedures in patients with longer anticipated survival is currently not known.

In selected patients with small aortic annuli at low surgical risk, SAVR with aortic root enlargement may represent a durable alternative, improving immediate hemodynamics and enabling the use of larger THV should redo interventions be required [107, 108].

### 4.4 Valve Expansion and Optimized First Implant

Symmetric and complete expansion of THVs appears to be critical for optimal leaflet mechanics, physiological flow patterns and long‐term valve performance. Incomplete THV expansion has been associated with impaired neosinus washout and hypoattenuated leaflet thickening (HALT) development and persistence [109, 110, 111], as well as an increased risk of hemodynamic valve deterioration at mid‐term follow‐up [112]. Valve asymmetric expansion has also been linked to poorer hemodynamics and a greater incidence of PVR [113, 114], while a post hoc analysis of the ACURATE IDE trial evidenced a nearly twofold increase in major adverse clinical events at 1 year with valve underexpansion [113]. Registry data evaluating the performances of last‐generation BEVs show similar rates of underexpansion and HALT with SAPIEN 3 Ultra RESILIA relative to prior‐generation devices, underscoring that this challenge persists despite iterative design refinements [115].

Annular and leaflet calcium burden directly correlate with THV expansion. Mechanical calcium‐modification therapies are currently under study. Dedicated leaflet‐scoring [116] devices and noninvasive transthoracic ultrasound therapy have demonstrated early safety and modest hemodynamic improvements in patients deemed at too high risk for TAVI, with CE approval recently granted for the latter [117, 118]. Although unlikely to replace AVR, these technologies may ultimately find an application in heavily calcified anatomies to optimize TAVI procedures.

Standardized fluoroscopic criteria for THV symmetric expansion have been proposed for the SAPIEN [114, 119] and ACURATE platform [113], which is no longer on the market. Remarkably, real‐time fluoroscopic assessment of underexpansion by use of the commissural post height method has been effective in predicting hemodynamic valve deterioration following TAVI with balloon‐expandable valves [112]. Post‐deployment assessment of THV stent frame expansion with intravascular ultrasound is also an emerging technology [120, 121], although larger evidence in its support is still awaited.

The decision to post‐dilate involves a balance between improving the hemodynamic outcome versus the risks of balloon post‐dilatation (BPD), as stroke, valve embolization, and new conduction abnormalities. Moreover, concerns exist about BPD inducing mechanical stress on the valve leaflets or on the stent frame, causing acute leaflet dysfunction or impaired leaflet durability through longitudinal micro‐cleavages and collagen fibril disruption [122]. Notwithstanding these concerns, recent registry data reinforce the acute clinical safety of BPD and suggest even lower incidence of valve dysfunction and bioprosthetic valve failure (BVF) at follow‐up [123, 124]. Routine BPD in small BEVs has recently been advocated, leading to an approximately 9% increase in minimal THV expansion by cross‐sectional area [125], lower incidence of PPM and comparable procedural success to selective post‐dilatation [126]. The ‘double‐tap’ technique—repeat BPD in BEVs using the same delivery balloon at unchanged volume—improves fluoroscopically measured expansion while limiting overexpansion risk, likely through reduced elastic recoil and additional calcium modification [125, 127]. Balloon selection matters: bench data in the Evolut FX+ confirm safety within IFU‐recommended sizes, whereas oversized balloons cause outward overexpansion and a spectrum of leaflet damage at suture sites [128]. The OptEx‐TAVI trial (NCT07042529) is currently evaluating whether systematic optimized pre‐ and post‐dilatation, compared to standard of care, can reduce the occurrence of HALT after TAVI.

**FIGURE 3:**
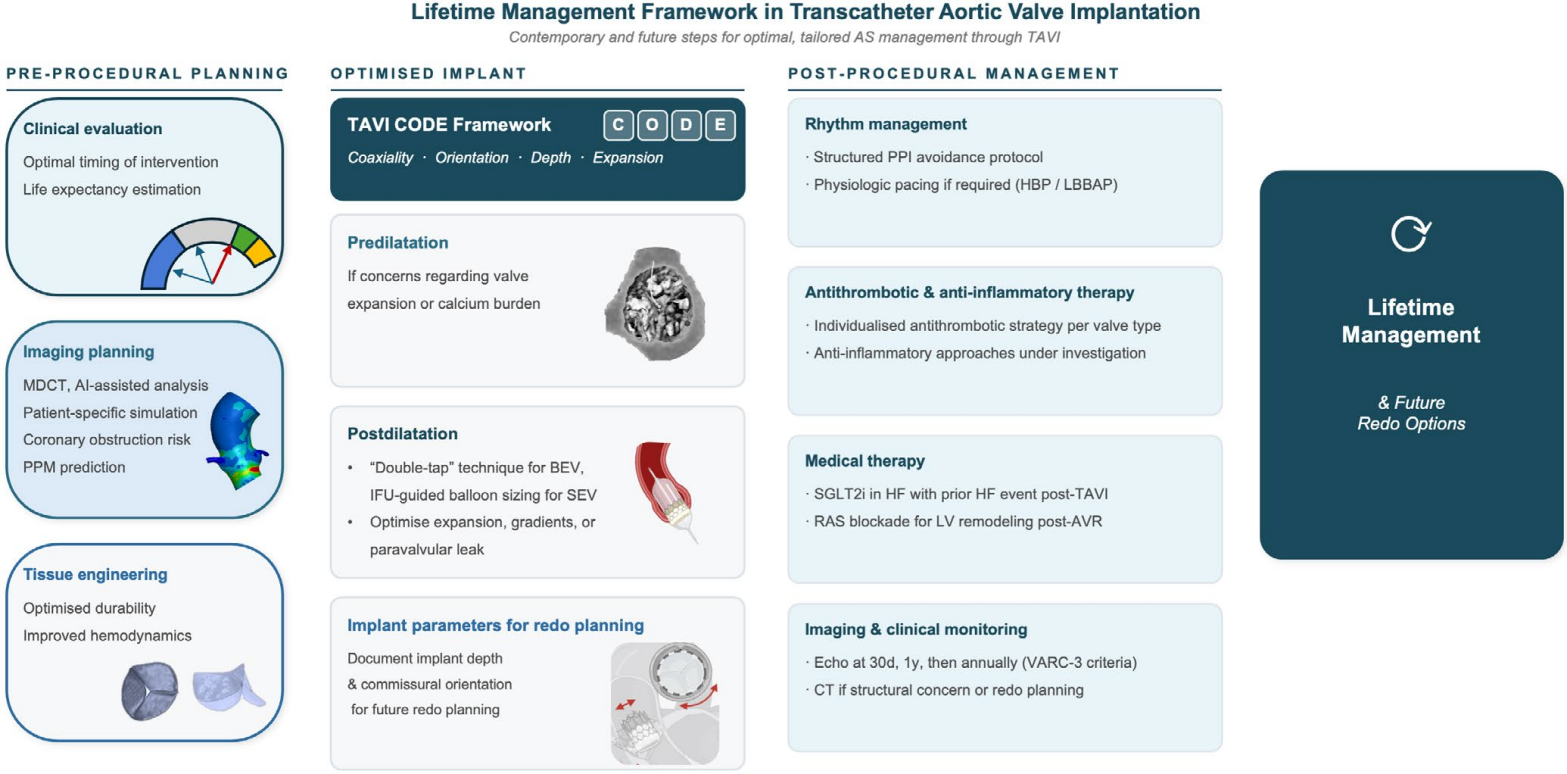
Lifetime management framework in transcatheter aortic valve implantation. Contemporary and future steps for optimal, tailored AS management through TAVI. AI, artificial intelligence; AS, aortic stenosis; CODE, Coaxiality, Orientation, Depth, Expansion; CT, computed tomography; HBP, His‐bundle pacing; HF, heart failure; LBBAP, left bundle branch area pacing; MDCT, multidetector CT; PPI, permanent pacemaker implantation; PPM, prosthesis–patient mismatch; RAS, renin–angiotensin system; SGLT2i, sodium–glucose cotransporter‐2 inhibitor; TAVI, transcatheter aortic valve implantation; VARC‐3, Valve Academic Research Consortium‐3.

Collectively, these considerations are encompassed within the recently proposed CODE framework—Coaxiality, Orientation (commissural alignment), implant Depth and Expansion—as a structured approach to optimal THV implantation and hemodynamic performance (Figure 3) [129, 130].

### 4.5 Procedure‐Related Embolization and Stroke

Clinically overt stroke has been reported to occur in a similar proportion of patients after TAVI and SAVR (~3% at 30 days, ~5% at 1 year) [131]. Stroke outcomes diverge sharply according to stroke severity. According to VARC‐3, stroke is classified as disabling if the acquired deficit corresponds to a modified Rankin Scale (mRS) score ≥ 2 at 90 days representing an increase of at least one category from the patient's pre‐stroke baseline [132]. This distinction matters most for prognosis: although recent CEPD trials report disabling stroke in only 0.5%–1.2% of patients [133, 134], a single‐center TAVI cohort found 30‐day and 1‐year mortality of 25% versus 0% and 40.6% versus 10.5% for disabling versus non‐disabling stroke, respectively [135].

Management of acute stroke complicating TAVI follows general stroke‐care principles, complicated by the recent large‐bore arterial access and by a persistent lack of dedicated guidelines and outcome data specific to this population [136, 137]. For moderate‐to‐severe strokes (defined by a National Institutes of Health Stroke Scale, NIHSS, score of ≥ 6 [132]) with large‐vessel or proximal medium‐vessel occlusion, with a favourable perfusion mismatch on neuroimaging, mechanical thrombectomy should be the treatment of choice [138]. Observational data suggest nearly 3‐fold higher odds of disability‐free survival at 90 days with neurointervention versus conservative management after adjustment for baseline severity [139]; however, thrombectomy may be technically more complex in this setting because of calcified emboli that standard devices cannot retrieve, increased vessel fragility and perforation risk, and coexisting chronic carotid disease. Thrombolysis, in turn, carries a disproportionate bleeding risk given recent large‐bore access and the frailty of this population [138]. In both scenarios, recognition may be delayed by general anaesthesia, post‐procedural delirium, and delayed mobilization, and depends on prompt on‐site neurointerventional availability; those considerations argue for a pre‐specified, multidisciplinary heart–brain stroke pathway at TAVI centers.

Cerebral embolic protection devices (CEPDs) were designed to capture embolic debris and prevent neurologic injury during the procedure. Observational and mechanistic studies confirmed their safety and ability to reduce new lesions on cerebral magnetic resonance imaging (MRI) [140, 141, 142], but the two large randomized trials to date have not shown a clinical stroke‐prevention benefit. In PROTECTED TAVI (3000 patients), CEPD use did not reduce all stroke within 72 h (2.3% vs. 2.9%, *p* = 0.30), though disabling stroke was numerically lower with CEPD (0.5% vs. 1.3%) [133]; BHF PROTECT‐TAVI (7601 patients) confirmed no difference in stroke within 72 h (2.1% vs. 2.2%) [134]. Neither trial showed benefit in any prespecified subgroup, including severe aortic calcification [143], bicuspid anatomy, prior stroke, sex, or surgical‐risk category, and a secondary analysis of BHF PROTECT‐TAVI found no effect of CEPD on post‐TAVI cognitive decline, the presumed downstream consequence of subclinical embolization [144]. Two recent meta‐analyses spanning both filter‐ and deflector‐based devices likewise confirm no benefit for any stroke, disabling stroke, new MRI‐detected lesions, or mortality [145, 146]. Real‐world registry data tell a more favourable story: in the STS/ACC TVT Registry (414,649 patients), CEPD use was associated with a reduction in disabling stroke by propensity‐weighted analysis (OR 0.79) and, more modestly, by instrumental‐variable analysis (RR 0.87), with the largest benefit in patients with prior stroke (disabling stroke here defined as in‐hospital stroke with death or non‐home discharge) [147]. This RCT–registry discordance likely reflects the anterior‐circulation‐only coverage of currently available filters (Sentinel; Boston Scientific), the low absolute periprocedural stroke rate in contemporary TAVI, and residual confounding by indication in registries. CEPDs also address only embolic stroke: hypoperfusion‐related border‐zone infarcts (particularly during rapid pacing) and the intrinsic thrombogenicity of the crimped native leaflets, neosinus, and bioprosthesis remain unprotected procedural and post‐procedural stroke mechanisms.

Routine CEPD use is therefore not supported by randomized evidence, though a role may remain in selected high‐risk scenarios (prior stroke, heavily calcified arch, leaflet‐modification procedures). Next‐generation devices extending protection to the posterior circulation and systemic embolization [148, 149, 150, 151, 152], and carbon‐dioxide flushing of the delivery system to reduce gaseous emboli [153], are in early feasibility testing but evidence from randomized trials is awaited.

### 4.6 Infective Endocarditis After Aortic Valve Implantation

Infective endocarditis (IE) after AVR is uncommon (Figure 2) but carries among the highest mortality of any post‐procedural complication, and its relevance is set to grow as TAVI is extended to younger, longer‐surviving patients. Reported incidence is broadly similar between TAVI and SAVR [154, 155, 156, 157, 158], lower with TAVI in some series [159, 160], and as low as 0.2% at 1 year in low‐risk trial populations [83, 84]; a ‘minimalist’ transfemoral approach has been associated with fewer very‐early, access‐ or procedural‐related infections [155].

IE in TAVI, however, poses distinct diagnostic and clinical challenges. Presentation is frequently atypical (fever in as few as 15%–80% of cases, versus ~90% in native or surgical‐valve IE) [161], and the modified Duke criteria perform poorly as a result. Echocardiographic assessment is hampered by the transcatheter frame, which can obscure vegetations or host them directly on the stent rather than the leaflets (in up to 34% of self‐expanding valves) [162, 163, 164]; computed tomography (CT) assessment might be challenged by leaflet thickening and obstructive patterns that can mimic valve thrombosis or degeneration; and 18F‐fluorodeoxyglucose positron emission tomography/CT may show non‐specific, homogeneous uptake from chronic sterile inflammation for up to 3 months post‐implant [165]. Given these pitfalls, a low threshold for transesophageal echocardiography and multimodality imaging is warranted whenever prosthetic‐valve IE is suspected [166, 167].

Once established, prognosis remains poor regardless of treatment strategy, with in‐hospital mortality ranging from 16% to 64% and 1‐year mortality from 27% to 75% across reported series [161].

Treatment remains predominantly medical: in a recent meta‐analysis, only 10% of patients with TAVI‐IE underwent surgical explantation, reflecting the advanced age, frailty and prohibitive surgical risk typical of this population [168]. Refinements in explantation techniques, including the Double Kocher, tourniquet, and Y‐incision aortic‐root enlargement approaches have improved the feasibility of removing a deeply seated, endothelialized transcatheter valves [169]. Together with the extension of TAVI to younger and lower‐risk patients, these advances argue against reflexively deferring surgery in otherwise reasonable operative candidates. Given the poor prognosis of IE regardless of treatment strategy, this complication should be factored into the lifetime risk profile of any aortic bioprosthesis, transcatheter or surgical.

### 4.7 Durability of TAVI and SAVR

Historically, durability of surgical bioprosthetic valves was derived from small observational series, frequently industry‐sponsored, in which durability was largely equated with freedom from reintervention [170]. This paradigm underestimated the true burden of valve failure, as it excluded patients not offered redo surgery or those dying with a dysfunctional valve. The adoption of standardized definitions by the Valve Academic Research Consortium‐3 (VARC‐3) has improved consistency across studies and clarified mechanisms of valve failure [132]. VARC‐3 distinguishes structural valve deterioration (SVD; intrinsic leaflet calcification, fibrosis, or tear), non‐structural valve dysfunction (e.g., underexpansion, paravalvular regurgitation, PPM), valve thrombosis, and endocarditis, all of which may culminate in BVF. Notably, VARC‐3 defines hemodynamic SVD as a change in valve hemodynamics relative to a stable early post‐procedural reference (increase in mean gradient ≥ 10 mmHg for stage 2 or ≥ 20 mmHg for stage 3), whereas the earlier definition by Capodanno et al. did not require longitudinal gradient change and may overestimate SVD by including PPM [171]. These methodological differences are critical when comparing durability outcomes across trials.

Durability data for surgical valves remain heterogeneous, derived from regulatory reports and observational cohorts with inconsistent endpoint definitions and variable follow‐up [170], as shown in Table 3. Long‐term analyses are further limited by competing mortality and inclusion of prior‐generation devices no longer in use. Although freedom from SVD is typically near 100% at 5 years, degeneration increases beyond 7–10 years. Notably, extended follow‐up contributed to the discontinuation of the Trifecta and Mitroflow valves due to earlier‐than‐expected structural degeneration, despite optimized hemodynamics at baseline.

**TABLE 3:** Durability data of contemporary surgical bio‐prostheses for SAVR.

	Reintervention for any cause	Freedom from SVD	Evidence in younger patients	Valve‐in‐valve considerations
Hancock II and Hancock II Ultra (Medtronic, Porcine Stented)	5Y: 1.5% 10Y: 3.5% 15Y: 4.9% [172]^,^ ^a^	10Y: 77.9%–97.6% 15Y: 61.6%–88.9% 20Y: 25%–63.4%	Freedom from SVD at 15Y: – 70% if < 60 years – 96.5% if > 60 years [173]	– Leaflet position: internal – Fracturable: No – Fluoroscopic marker: sewing ring and stent post tip
Mosaic and Mosaic Ultra (Medtronic, Porcine Stented)	5Y: 1.5% 10Y: 3.5% 15Y: 4.9% [172]^,^ ^a^	5Y: 96.2% 10Y: 87.1%–100%	Freedom from SVD at 12Y: – 93.5% if < 65 years – 98.2% if > 65 years [174] Reintervention for SVD at 17Y: – 52.4% if < 60 years – 19% if > 60 years [175]	– Leaflet position: internal – Fracturable: Yes – Fluoroscopic marker: stent post tip
Biocor Epic—Epic Max and Epic Plus (Abbott, Porcine Stented)	5Y: 2.0% 10Y: 3.7%–2.6% 15Y: 4.7% [172, 176]	5Y: 98.4%–99.6% 10Y: 93.1%–99.3% 15Y: 88.4%–95.6% 20Y: 70.3% [177]		– Leaflet position: internal – Fracturable: Yes – Fluoroscopic marker: sewing ring and stent post tip
Perimount – Perimount 2900, Magna, Magna Ease (Edwards Lifesciences, Stented Bovine Pericardial)	5Y: 1.3%–1.5% 10Y: 3.0%–5.0% 15Y: 5.1%–13.3% [172, 178]	5Y: 100% 10Y: 86%–98.1% 15Y: 78.6%–87.7% 20Y: 85%	Freedom from SVD if < 65 years: – 90% at 10Y – 72% at 15Y – 48% at 20Y [179] – 93% at 12Y with CE Magna Ease [180]	– Leaflet position: internal – Expandable – Fluoroscopic marker: stent
INSPIRIS RESILIA (Edwards Lifesciences, Stented Bovine Pericardial)	5Y: 1.3% [181]	5Y: 100% 7Y: 99.3% [182]	No data	– Leaflet position: internal – Expandable – Fluoroscopic marker: stent
Avalus and Avalus Ultra (Medtronic, Stented Bovine pericardial)	7Y: 5.7% [183]	100% at 5 years 98.8% at 7 years [183]	No data	– Leaflet position: internal – Fracturable: No – Fluoroscopic marker: stent
Freestyle – Full root, complete subcoronary or modified subcoronary (Medtronic, Porcine Stentless)	Reintervention for SVD: – Subcoronary: at 10Y 5.4%; at 15Y 23.3% – Full root: at 1.1% 10Y; at 15 V 11.9% [184]	10Y: 97%	Reintervention for SVD: – 10Y 2.2% and 15Y 29.3% if < 60 years – 10Y 3.8% and 15Y 13.5% if > 60 years [185]	– Pocine aortic root, sewing ring at the base or sutured leaflets inside the root – Fracturable: NA – Fluoroscopic marker: none
Freedom SOLO (Corcym‐Sorin, Stentless Bovine Pericardial)	7Y: 6% [186]	5Y: 92%–100% 10Y: 90.8%	No data	– Sutured inside the root – Fracturable: NA – Fluoroscopic marker: none
Pericarbon Freedom (Corcym‐Sorin, Equine Pericardial Stentless)	13Y: 7.7% [187]	5Y: 100% 10Y: 83.9%–96% 13Y: 92.3%	No data	– Sutured inside the root – Fracturable: NA – Fluoroscopic marker: none
Perceval (Corcym‐Sorin, Bovine Pericardial Sutureless)	5Y: 97% 10Y: 94% [188]	5Y: 95.5%	No data	– Leaflet position: internal – Expandable – Fluoroscopic marker: stent
EDWARDS INTUITY (Edwards Lifesciences, Bovine pericardial rapid‐deployment valve)	No data	5Y: 99.6%–99.8% 10Y: 90.8% [189, 190]	No data	– Leaflet position: internal – Fracturable: Yes – Fluoroscopic marker: stent and skirt
Trifecta and Trifecta GT (Abbott, Pericardial leaflets) No longer on the market	7Y: 3.6% [191] 8Y: 16.9% [192]	5Y: 100%–95.5% 8Y: 70.6% [193]	No data	– Leaflet position: external – Fracturable: No – Fluoroscopic marker: stent
Mitroflow (Bovine Pericardial Stented) No longer on the market	5Y: 3.5% 10Y: 9.2% 15Y: 9.8% [172]	5Y: 94.7%–99.6% 10: 39.2%–92.5% 15Y: 5%–84.8%	No data	– Leaflet position: external – Fracturable: Yes – Fluoroscopic marker: sewing ring

Abbreviations: NA, not assessed; SVD, structural valve deterioration; Y, years.

^a^ Data referred to both Hancock and Mosaic.

Randomized trials and propensity‐matched registries now provide comparative durability data between TAVI and SAVR up to 5 years. In high‐ and intermediate‐risk populations, pooled 5‐year data from CoreValve High Risk and SURTAVI demonstrated lower cumulative incidence of VARC‐3–defined hemodynamic SVD after TAVI compared with surgery (2.2% vs. 4.4%) [194]. Among BEVs, 5‐year analyses from PARTNER 2A and the SAPIEN 3 registry highlight clear generation effects, with higher rates of hemodynamic SVD in SAPIEN XT (9.5%) compared with SAPIEN 3 (3.9%), rates broadly comparable to SAVR (3.5%) [195]. The recently published 10‐year outcomes from PARTNER 2A now extend this picture [196], revealing late divergence: all‐cause mortality was significantly higher after TAVI with the SAPIEN XT compared with SAVR (86.1% vs. 82.8%; *p* = 0.02), with reintervention rates also significantly elevated (6.3% vs. 1.6% after competing risk adjustment; *p* < 0.001), predominantly driven by restenosis and performed almost exclusively via valve‐in‐valve TAVI. In contrast, the propensity‐matched 10‐year analysis of the PARTNER 2 SAPIEN 3 Intermediate‐Risk Registry [197] found no significant difference in mortality (83.4% vs. 82.3%) or reintervention (2.0% vs. 1.9%), with regurgitation rather than stenosis as the predominant failure mode, reinforcing the impact of computed tomography‐based pre‐procedural planning and the generation effect already evident at 5 years [195].

In low‐risk populations, follow‐up now extends beyond 5 years. In PARTNER 3, cumulative incidence of BVF at 7 years was similar between TAVI and SAVR (6.9 vs. 7.5%), with similar rates of reintervention, valve‐related death, and stage 3 hemodynamic valve deterioration [198]. The Evolut Low Risk similarly demonstrated comparable survival and valve‐related outcomes between TAVI and SAVR through 6 years. However, reintervention rates diverged beyond year 5, becoming significantly higher after TAVI at 7 years (9.8% vs. 6.0%; sHR 1.68 [95% CI 1.10–2.58]). This difference was largely driven by regurgitation‐related rather than stenosis‐related reinterventions and was predominantly observed among recipients of larger devices, suggesting a contribution of device design and procedural factors [199]. At 10 years, NOTION reported lower severe SVD with TAVI (1.5% vs. 10%), and numerically lower BVF (9.7% vs. 13.8%) [200].

Cross‐trial comparisons nevertheless remain constrained by competing mortality, incomplete imaging adjudication, device and era heterogeneity, and non‐uniform endpoint definitions. Table 4 presents single‐platform durability data across randomized trials and registries. Whether durability differences represent a class effect or reflect specific valve design and generation remains unresolved.

**TABLE 4:** Durability data of transcatheter bioprostheses^a^.

Valve model	Surgical risk	Aortic valve reintervention	Moderate or severe SVD	BVF	Type of studies
SAPIEN 1st Generation [201, 202]	Inoperable or high	5Y: 0.0%	NA	NA	RCT
SAPIEN XT [195, 203, 204]	Intermediate or high	5Y: 2.5%–3.2% 10Y: 6.3%	5Y: 6.5%–9.5%	5Y BVF: 4.1%–4.7%^b^	RCT
SAPIEN 3 [195, 205]	Intermediate or low	5Y: 2.6% (low‐risk) 7Y: 6.7% (low risk) 10Y: 2.0% (intermediate risk)	5Y: 3.9%	5Y: 1.3%–3.3%^b^ 7Y: 6.9%^b^	RCT and prospective registries
Corevalve [41, 200, 206]	Extreme to low	5Y: 0.6%–3.0% 10Y: 4.3%	5Y: 0%–10%^c^ 8Y: 4.6% 10Y: 15.4%	5Y: 3.4% 10Y: 9.7%	RCT and prospective registries
Evolut R/Pro [199]	Low	5Y: 3.3% 6Y: 5.5% 7Y: 9.8%	NA	NA	RCT
Portico/Navitor valve [207]	High	5Y: 2.0%	5Y: 0.9%	5Y: 2.7%	Prospective registries
Myval [208]	Intermediate	4Y: 0.0%	4Y stage 2 or 3 HVD: 10.4^d^	4Y: 3.3%	Single ambispective registry
VitaFlow (PCRLV 2023, D. Zhou)	High	5Y: 1.0% 7Y: 1.25%	7Y: 8.1%	7Y: 3.8%	Prospective registry
Acurate Neo [209, 210]	Intermediate	5Y: 1.1%	39 m stage 2 or 3 HVD: 2.2%	39 m: 1.4%	Prospective registry

Abbreviations: BVF, bioprosthetic valve failure; HVD, hemodynamic valve deterioration; NA, not assessed; RCT, randomized control trial; Y, years.

^a^ These data only comprise evidence regarding a single, specific THV platform. Table S1 gives a larger perspective of long‐term TAVI durability comprising studies evaluating multiple platforms.

^b^ Using VARC‐3 definition [211].

^c^ According to EAPCI/EACTS definition [171].

^d^ No available data on SVD.

Beyond the generation effect among BEVs [195], newer‐generation self‐expanding Evolut valves show lower 5‐year reintervention rates than first‐generation CoreValve, with differences clustering in the first year, thus suggesting a significant contribution of procedural factors such as sizing, implantation technique and sealing technology [212]. Analyses of TAVI explants and redo procedures have revealed distinct failure patterns across valve types: SVD predominates in balloon‐expandable valves, while non‐structural dysfunction, particularly paravalvular regurgitation, is more common with self‐expanding platforms [213].

Computational modelling offers mechanistic insight into SVD beyond conventional imaging. Patient‐specific simulation can quantify surrogate metrics like leaflet stress, neo‐sinus washout, and thrombogenic flow shaped by anatomy and TAVI‐specific mechanical insults including crimping, asymmetric expansion and underexpansion [214, 215, 216, 217, 218]. Current models remain constrained by idealized geometries and limited validation cohorts, but hold potential to support risk stratification, guide device selection and inform prosthesis design.

### 4.8 Redo Strategies After TAVI and SAVR

Lifetime management requires realistic estimation of life expectancy at the time of index intervention. Population data suggest persistent loss of life expectancy after SAVR, particularly in younger patients. In SWEDEHEART, patients undergoing SAVR experienced an estimated 1.9‐year reduction in life expectancy overall, with a pronounced age interaction: loss of life expectancy was greatest in younger patients (< 50 years, approximately 4.4 years) and minimal in the very elderly (> 80 years, approximately 0.4 years) [219]. Survival is also strongly influenced by baseline surgical risk, with median survival approximately 10.9 years in low‐risk, 7.3 years in intermediate‐risk and approximately 5.8 years in high‐risk patients [220]. Whether these findings translate to contemporary TAVI populations remains uncertain. Aligning anticipated survival with expected valve durability is essential for surveillance planning and redo strategy selection.

Reintervention strategies after surgical bioprosthesis failure include valve‐in‐valve TAVI (ViV‐TAVI) and redo surgery. Compared with redo surgical AVR, ViV‐TAVI is consistently associated with lower early morbidity, including reduced bleeding, acute kidney injury and shorter recovery time, as well as lower early mortality [221, 222, 223, 224, 225, 226, 227]. However, some observational data suggest possible convergence or reversal of outcomes favouring redo SAVR beyond 1–2 years. The REPEAT trial (DRKS00034170) will further clarify outcomes in younger, lower‐risk patients.

For failed TAVI, redo‐TAVI and surgical explant are the principal strategies. Although the individual risk of reintervention is low, its absolute burden is increasing with expanding TAVI volumes: in a large Medicare cohort of more than 400,000 procedures, 0.9% required reintervention, with redo‐TAVI accounting for nearly two‐thirds of cases and surgical explant for approximately one‐third, the latter driven largely by infective endocarditis [228]. Surgical explant of THVs carries substantially higher perioperative risk than redo SAVR, with in‐hospital mortality exceeding 10% in contemporary registries [229, 230], and frequently requires concomitant procedures that further increase complexity [231]. Redo‐TAVI, while less invasive, is anatomically precluded in a meaningful proportion of patients (26.8% of patients in the EXPLANT‐TAVR registry) [229, 230] and may be unfavourable in the setting of severe PPM, high residual gradients, or coronary obstruction risk.

The procedural planning and technical execution of ViV‐TAVI and redo‐TAVI are areas of rapidly evolving complexity, encompassing leaflet modification techniques, coronary protection strategies, and device‐specific implantation sequences that merit dedicated review beyond the scope of this paper.

What this review does wish to emphasize is that the feasibility and safety of future reinterventions are substantially determined by decisions made at the index procedure: valve type and size, implant depth, commissural alignment and meticulous CT‐based planning (Figure 2).

Emerging tools, including dedicated apps for THV selection [232], standardized redo‐TAVI planning protocols, and defined procedural guidance for first‐to‐second THV combinations [233], are expanding the boundaries of what is technically achievable in anatomically challenging patients.

Ultimately, THV durability and redo feasibility are not separate concerns but integral components of a unified lifetime valve management strategy. Progress will require coordinated advances in biomaterials, device design, and leaflet technologies [234, 235] alongside a clinical culture that treats optimal initial implantation and preservation of future options as the primary goal of every intervention.

## 5 Conclusions

Aortic stenosis is a progressive disease whose outcomes are shaped by the timing of diagnosis, the decision to intervene, and the quality of the index procedure. Randomized evidence now supports earlier intervention in asymptomatic severe AS, with growing recognition that watchful waiting may expose patients to silent myocardial injury and abrupt decompensation. The stable/progressive/acute valve syndrome framework provides a clinically actionable basis for decision‐making, and ongoing trials in moderate AS may extend the therapeutic window further upstream.

As TAVI expands to younger patients, procedural quality assumes paramount importance. The CODE framework encapsulates the technical imperatives of optimal implantation, and comparable mid‐term durability between TAVI and SAVR has been established in low‐risk populations, though diverging reintervention rates beyond 5–7 years underscore the need for lifetime management planning—encompassing surveillance, redo feasibility, and preservation of future treatment options.

Several important gaps remain. No pharmacologic therapy has yet altered valve disease progression, though targeted lipid‐lowering and anti‐inflammatory approaches are under active investigation. Artificial intelligence–assisted imaging analysis for AS diagnosis and procedural planning, together with computational modelling tools and multi‐omic characterization of AS, is rapidly evolving but still requires prospective clinical validation. We envision that decisions on timing of intervention will increasingly rest on a combined strategy integrating multiple domains, rather than on clinical and echocardiographic grading alone. Addressing these gaps through coordinated trials and multidisciplinary collaboration will be essential to realizing a truly lifetime‐oriented approach to aortic stenosis care.

## Funding

The authors received no financial support for the research, authorship, and/or publication of this article.

## Disclosure

Dr. Pilgrim reports research grants from the Swiss National Science Foundation, the Swiss Heart Foundation, the Swiss Polar Institute, the Bangerter‐Rhyner Foundation, the Mach‐Gaensslen Foundation, and the Monsol Foundation. Research, travel or educational grants to the institution without personal remuneration from Biotronik, Boston Scientific, Edwards Lifesciences, and ATSens; speaker fees and consultancy fees to the institution from Biotronik, Boston Scientific, Edwards Lifesciences, Abbott, Medtronic, Biosensors, and Highlife. Dr. Cozzi has no relationships relevant to the contents of this article to disclose.

## Ethics Statement

The authors have nothing to report.

## Conflicts of Interest

The authors declare no conflicts of interest.

## Supporting information


**Table S1:** Medium‐ and long‐term studies evaluating durability of TAVR Table S1. Medium‐ and long‐term studies evaluating durability of TAVR. BEV = balloon expandable valve, BVD = bioprosthetic valve dysfunction, BVF = bioprosthetic valve failure, CI = confidence interval, HVD = hemodynamic valve deterioration, IA = intra‐annular, pts. = patients, RCT = randomized control trial, SA = supra‐annular, SEV = self‐expanding valve, STS = Society of Thoracic Surgeons Predicted Risk of Mortality score, SVD = structural valve deterioration, * Using VARC‐3 definition^27^† according to EAPCI/EACTS definition^28^.

## Data Availability

No datasets were generated or analysed during the current study. All information discussed in this review is derived from previously published studies, which are cited in the reference list.
