# Supplementary material for: Contemporary Management of Aortic Stenosis: Timing of Intervention and Lifetime Valve Management
Source: Eur J Clin Invest. 2026 Aug 13;56(8):e70245. doi: 10.1111/eci.70245 (PMC13473407; doi:10.1111/eci.70245)
Supplement: Supplementary file 1 [file ECI-56-e70245-s001.docx]

**Supplementary Table s1. Medium- and long-term studies evaluating durability of TAVR**

| **STUDY TYPE** | **STUDY NAME** | **THV type** | **Max follow-up** | **Mortality** | **Reintervention** | **Structural Valve deterioration** |
| --- | --- | --- | --- | --- | --- | --- |
| RCT  Inoperable | **PARTNER 1 Extreme Risk**^1^  (N=165) | SAPIEN 1^st^ generation | 5 years | 71.8% | 0.0% |  |
| Single arm-trail  Inoperable | **CoreValve US Extreme Risk Pivotal**^2^  (N=639) | Corevalve | 5 years | 71.6% | 2.9% (1.0-7.8%)  out of 158 pts |  |
| RCT  High risk | **PARTNER 1 High Risk**^3^  (N=348) | SAPIEN 1^st^ generation | 5 years | 67.8% | 0.0% |  |
| RCT  High risk | **CoreValve US High Risk Pivotal**^4^  (N=391) | Corevalve | 5 years | 55.3% | 10/390 (3.0%) | Moderate SVD: 36/390 (9.2%)  Severe SVD: 3/390 (0.8%)$\dagger$ |
| RCT  High risk | **CHOICE**^5^  (N=241) | SAPIEN XT  CoreValve | 5 years | 48.5% | 3 pts (2.5%) BEV  3 pts (2.6%) SEV | Moderate SVD: 4 pts (5.6%) with BEV, 0.0% SEV  Severe SVD: 2 pts (0.9%) with BEV, 0.0% SEV  BVF: 6 pts (4.1% with BEV), 4 pts (3.4%) with SEV |
| RCT  Intermediate risk | **PARTNER 2A**^67^  (N=974; 774 with 30-day echo assessment) | SAPIEN XT | 5 years | 46% | 3.2% | SVD: 44 pts; 9.5% (7.0-12.%)  SVD-related BVF: 16 pts; 3.7% (0.5-2.5%)  All-cause BVF: 25 pts, 4.7% (3-1-7.1%)* |
| RCT  Intermediate risk | **SURTAVI**^8^  (N=864) | Corevalve 84%  Evolut R 16% | 5 years | 30% | 27 (3.5%) | SVD: 1.82% in a pooled analysis with Corevalve US HR^9^* |
| RCT  Low risk | **PARTNER 3**^10^  (N=496) | SAPIEN 3 | 7 years | 19.5% | 28 (6.7%) | BVF 5 Y 3.3%*  BVF 7 Y 6.9% *  Stage 3 HVD: 1.7% |
| RCT  Low risk | **Evolut LR**^11^  (N= 730) | Corevalve, Evolut R, Evolut Pro | 6 years (prespecified)  7 years (exploratory) | 6Y: 23.3% | 6Y: 5.5%  7Y: 9-8% | NA |
| RCT  Intermediate to low risk | **NOTION**^12^  **(**N= 145) | Corevalve | 10 years | 62.7% | 4.3% | BVF: 9.7%  Moderate o more SVD: 15.4%  Severe SVD: 1.5%*  Severe BVD: 20.5% |
| Multicentric registry  High risk | **Barbanti et al, 2015**^13^  **(**N= 353) | Corevalve | 5 years | 55% | 2 pts, 0.6% | Late prosthesis failure: 5 patients, 1.4% (VARC-1) |
| Multicentric registry  High risk | **FRANCE-2 registry**^14^  **(**N= 4210) | SAPIEN or SAPIEN XT 66.0%  CoreValve 33.7% | 5 years  (N patients for echo SVD analysis = 459) | 60.8% |  | Moderate SVD BEV: 27/195 (13.8%)  Severe SVD BEV: 8/195 (4.1%)  Moderate SVD SEV: 7/79 (8.9%)  Severe SVD SEV: 0/79 (0.0%)† |
| Multicentric registry  High risk | **Orvin et al, 2019**^15^  **(**N=450) | BEV and SEV | 5 years | 59.1% |  | Severe SVD 22/184 (12.3%)  BVF: 6/184(3.3%)† |
| Multicentric registry  Intermediate risk | **SAPIEN 3 intermediate-risk**^6^  (N=1069) | SAPIEN 3 | 5 years  N=891 |  |  | SVD: 22 pts, 3.9 % (2.5-6.0%)  SVD-related BVF: 6 pts, 1.1% (0.5-2.5%)  BVF: 13 pts, 1.3% (0.6-2.7%)* |
| Multicentric registry  High risk | **UK-TAVI registry**^16^  (N=241) | SAPIEN 19.3%, SAPIEN XT 15%, CoreValve 64%, Portico 1.7% | 5.8 Years | 67.8% |  | Moderate SVD 21/241 (8.7%) Severe SVD: 1 pts (0.4%)$\dagger$ |
| Monocentric registry  High risk | **Deutsch et al, 2018**^17^  (N=300) | SAPIEN 28.7%, CoreValve 71.3% | 5.8 Years | 75.6% |  | Any SVD 14.9% (CoreValve 11.8%; SAPIEN 22.6%)  Late BVF in 11 pts. $\dagger$ |
| Multicentric registry  High risk | **Durand et al, 2019**^18^  (n=1403) | BEV 85.8%, SEV 14.2% | 7 Years | 81.4% | 5 pts, 1% (95% CI 0.4-1.6%) | Moderate SVD 32/589  Severe SVD 17/589  BVF 19/589 (1.9%, 5% CI 1.4-2.4%)$\dagger$ |
| Monocentric registry  High risk | **Eltchaninoff et al, 2018**^19^  (N=378) | SEV or BEV | 8 Years | 90.4% |  | Moderate or severe SVD: 9 (3.2%; 95% CI 1.45-6.11%)  BVF: 2 (0.58%, 95% CI 0.15-2.75%)$\dagger$ |
| Monocentric registry  High risk | **Holy et al, 2018**^20^  (N=152) | Corevalve | 8.9 Years | 73% | 5 (3.3%) | BVF: 7.9%$\dagger$ |
| Multicentric registry  High risk | **Barbanti et al, 2018**^21^  (N=288) | SAPIEN XT 16.7%, CoreValve 82.6% | 8 Years | 58.7% | 2 (0.7%) | Severe SVD: 7 pts, 2.39%, 95% CI 0.77-5.71%)  BVF: 11 pts 4.51%, 95% CI 1.95-8.76%)$\dagger$ |
| Multicentric registry  High risk | **Testa et al, 2020**^22^  (N=990) | CoreValve | 8 Years | 73.8% |  | Moderate SVD: 3.0%, 95% CI 2.1-4.3%  Severe SVD: 1.6%, 95% CI 0.6-3,9%  Late BVF 2.5%, 95% CI 1.2-5% $\dagger$ |
| Monocentric registry  Moderate and high risk | **BERN-TAVI registry, 2025**^23^ (N=2403) | BEV 57%, SEV 39.5%, mechanical expandable 4.0% | 10 years |  |  | Moderate HVD: 120 pts, 5%  Severe HVD 46, 1.9%* |
| Multicentric registry and RCT  High risk | **PORTICO IDE, post-market, continued access** ^24^  (N=1464) | Portico | 5 Years | 44.3% | 2.0% | Moderate HVD: 0.9%  Severe HVD: 0.0%  BVF: 2.7%* |
| Multicentric registry  Median STS 4.8% | **PUREVALVE Registry**^25^  (N= 604) | 80.1% IA (SAPIEN, SAPIEN XT, SAPIEN 3, LOTUS); 19.9% SA (Corevalve, Evolut R, Acurate) | 10 Years |  |  | Moderate HVD: 7%  Severe HVD: 4%  BVF :6%* |
| Multicentric registry | **Silva I et al, 2025**  (N=2848) | 973 BEV and 875 SEV | 8 Years |  |  | Moderate or severe HVD: 11.5% (95% CI 8.9-14.9%)  BVF: 13.3% (95% CI 9.8-18.0%)* |
| Multicentric registry  Mean STS 5.0% | **European Valve Durability TAVI Registry**^26^  (N=597) | Corevalve 51.2%, SAPIEN XT 39.9%, Lotus 5.0%, Direct flow 2.7%, portico 0.7%, Evolut R 0.5% | Median FU 6.1 Years |  |  | Moderate SVD in 37 pts 6.2%; severe SVD: 20 pts, 3.4%)  Moderate or severe SVD: 5.4% (95% CI 3.0-7.7) at 7Y, 33.2% (95% CI 23.6-41.6%) at 10 years |

Table s1. Medium- and long-term studies evaluating durability of TAVR. BEV= balloon expandable valve, BVD= bioprosthetic valve dysfunction, BVF = bioprosthetic valve failure, CI= confidence interval, HVD = hemodynamic valve deterioration, IA= intra-annular, pts= patients, RCT= randomized control trial, SA= supra-annular, SEV= self-expanding valve, STS = Society of Thoracic Surgeons Predicted Risk of Mortality score, SVD= structural valve deterioration, * Using VARC-3 definition^27^† according to EAPCI/EACTS definition^28^

**References**

1.	Kapadia SR, Leon MB, Makkar RR, et al. 5-year outcomes of transcatheter aortic valve replacement compared with standard treatment for patients with inoperable aortic stenosis (PARTNER 1): a randomised controlled trial. *Lancet*. 2015;385(9986):2485-2491. doi:10.1016/S0140-6736(15)60290-2

2.	Arnold S V, Petrossian G, Reardon MJ, et al. Five-Year Clinical and Quality of Life Outcomes From the CoreValve US Pivotal Extreme Risk Trial. *Circ Cardiovasc Interv*. 2021;14(6):e010258. doi:10.1161/CIRCINTERVENTIONS.120.010258

3.	Mack MJ, Leon MB, Smith CR, et al. 5-year outcomes of transcatheter aortic valve replacement or surgical aortic valve replacement for high surgical risk patients with aortic stenosis (PARTNER 1): a randomised controlled trial. *Lancet*. 2015;385(9986):2477-2484. doi:10.1016/S0140-6736(15)60308-7

4.	G. GT, J. RM, J. PJ, et al. 5-Year Outcomes of Self-Expanding Transcatheter Versus Surgical Aortic Valve Replacement in High-Risk Patients. *JACC*. 2018;72(22):2687-2696. doi:10.1016/j.jacc.2018.08.2146

5.	Abdel-Wahab M, Landt M, Neumann F-J, et al. 5-Year Outcomes After TAVR With Balloon-Expandable Versus Self-Expanding Valves: Results From the CHOICE Randomized Clinical Trial. *JACC Cardiovasc Interv*. 2020;13(9):1071-1082. doi:10.1016/j.jcin.2019.12.026

6.	Philippe P, Julien T, A. JW, et al. Structural Deterioration of Transcatheter Versus Surgical Aortic Valve Bioprostheses in the PARTNER-2 Trial. *JACC*. 2020;76(16):1830-1843. doi:10.1016/j.jacc.2020.08.049

7.	Makkar RR, Thourani VH, Mack MJ, et al. Five-Year Outcomes of Transcatheter or Surgical Aortic-Valve Replacement. *N Engl J Med*. 2020;382(9):799-809. doi:10.1056/NEJMoa1910555

8.	Van Mieghem NM, Deeb GM, Søndergaard L, et al. Self-expanding Transcatheter vs Surgical Aortic Valve Replacement in Intermediate-Risk Patients: 5-Year Outcomes of the SURTAVI Randomized Clinical Trial. *JAMA Cardiol*. 2022;7(10):1000-1008. doi:10.1001/jamacardio.2022.2695

9.	O’Hair D, Yakubov SJ, Grubb KJ, et al. Structural Valve Deterioration After Self-Expanding Transcatheter or Surgical Aortic Valve Implantation in Patients at Intermediate or High Risk. *JAMA Cardiol*. 2023;8(2):111-119. doi:10.1001/jamacardio.2022.4627

10.	Leon MB, Mack MJ, Pibarot P, et al. Transcatheter or Surgical Aortic-Valve Replacement in Low-Risk Patients at 7 Years. *N Engl J Med*. October 2025. doi:10.1056/NEJMoa2509766

11.	K. FJ, J. YS, Michael DG, et al. Six-Year Outcomes After Transcatheter vs Surgical Aortic Valve Replacement in Low-Risk Patients With Aortic Stenosis. *JACC*. 2026;0(0). doi:10.1016/j.jacc.2026.02.5063

12.	Thyregod HGH, Jørgensen TH, Ihlemann N, et al. Transcatheter or surgical aortic valve implantation: 10-year outcomes of the NOTION trial. *Eur Heart J*. 2024;45(13):1116-1124. doi:10.1093/eurheartj/ehae043

13.	Barbanti M, Petronio AS, Ettori F, et al. 5-Year Outcomes After Transcatheter Aortic Valve Implantation With CoreValve Prosthesis. *JACC Cardiovasc Interv*. 2015;8(8):1084-1091. doi:10.1016/j.jcin.2015.03.024

14.	Didier R, Eltchaninoff H, Donzeau-Gouge P, et al. Five-Year Clinical Outcome and Valve Durability After Transcatheter Aortic Valve Replacement in High-Risk Patients. *Circulation*. 2018;138(23):2597-2607. doi:10.1161/CIRCULATIONAHA.118.036866

15.	Orvin K, Zekry S Ben, Morelli O, et al. Long-Term Functional and Structural Durability of Bioprosthetic Valves Placed in the Aortic Valve Position via Percutaneous Rout in Israel. *Am J Cardiol*. 2019;124(11):1748-1756. doi:10.1016/j.amjcard.2019.08.043

16.	Blackman DJ, Saraf S, MacCarthy PA, et al. Long-Term Durability of Transcatheter Aortic Valve Prostheses. *J Am Coll Cardiol*. 2019;73(5):537-545. doi:https://doi.org/10.1016/j.jacc.2018.10.078

17.	Deutsch M-A, Erlebach M, Burri M, et al. Beyond the five-year horizon: long-term outcome of high-risk and inoperable patients undergoing TAVR with first-generation devices. *EuroIntervention J Eur Collab with Work Gr Interv Cardiol Eur Soc Cardiol*. 2018;14(1):41-49. doi:10.4244/EIJ-D-17-00603

18.	Durand E, Sokoloff A, Urena-Alcazar M, et al. Assessment of Long-Term Structural Deterioration of Transcatheter Aortic Bioprosthetic Valves Using the New European Definition. *Circ Cardiovasc Interv*. 2019;12(4):e007597. doi:10.1161/CIRCINTERVENTIONS.118.007597

19.	Eltchaninoff H, Durand E, Avinee G, et al. Assessment of structural valve deterioration of transcatheter aortic bioprosthetic balloon-expandable valves using the new European consensus definition. *EuroIntervention*. 2018;14(3):e264-e271. doi:10.4244/EIJ-D-18-00015

20.	Holy EW, Kebernik J, Abdelghani M, et al. Long-term durability and haemodynamic performance of a self-expanding transcatheter heart valve beyond five years after implantation: a prospective observational study applying the standardised definitions of structural deterioration and valve failure. *EuroIntervention J Eur Collab with Work Gr Interv Cardiol Eur Soc Cardiol*. 2018;14(4):e390-e396. doi:10.4244/EIJ-D-18-00041

21.	Barbanti M, Costa G, Zappulla P, et al. Incidence of Long‐Term Structural Valve Dysfunction and Bioprosthetic Valve Failure After Transcatheter Aortic Valve Replacement. *J Am Heart Assoc*. 2018;7(15):e008440. doi:10.1161/JAHA.117.008440

22.	Testa L, Latib A, Brambilla N, et al. Long-term clinical outcome and performance of transcatheter aortic valve replacement with a self-expandable bioprosthesis. *Eur Heart J*. 2020;41(20):1876-1886. doi:10.1093/eurheartj/ehz925

23.	Bashir A, Daijiro T, Masaaki N, et al. Hemodynamic Valve Deterioration After Transcatheter Aortic Valve Replacement. *JACC Cardiovasc Interv*. 2025;18(1):72-85. doi:10.1016/j.jcin.2024.09.039

24.	Puri R, Thiele H, Fichtlscherer S, et al. Five-Year Clinical Outcomes and Durability of a Self-Expanding Transcatheter Heart Valve With Intra-Annular Leaflets. *Circ Cardiovasc Interv*. 2025;18(12):e015430. doi:10.1161/CIRCINTERVENTIONS.125.015430

25.	Scotti A, Fovino LN, Coisne A, et al. 10-Year Impact of Transcatheter Aortic Valve Replacement Leaflet Design (Intra- Versus Supra-Annular) in Mortality and Hemodynamic Performance. *Front Cardiovasc Med*. 2022;9:924958. doi:10.3389/fcvm.2022.924958

26.	Giannini C, Capodanno D, Toth GG, et al. Long-term structural valve deterioration after TAVI: insights from the EORP ESC Valve Durability TAVI Registry. *EuroIntervention*. 2025;21(10):537-549. doi:10.4244/EIJ-D-24-00662

27.	Généreux P, Piazza N, Alu MC, et al. Valve Academic Research Consortium 3: Updated Endpoint Definitions for Aortic Valve Clinical Research. *J Am Coll Cardiol*. 2021;77(21):2717-2746. doi:10.1016/j.jacc.2021.02.038

28.	Capodanno D, Petronio AS, Prendergast B, et al. Standardized definitions of structural deterioration and valve failure in assessing long-term durability of transcatheter and surgical aortic bioprosthetic valves: a consensus statement from the European Association of Percutaneous Cardiovascular Interventions (EAPCI) endorsed by the European Society of Cardiology (ESC) and the European Association for Cardio-Thoracic Surgery (EACTS). *Eur Heart J*. 2017;38(45):3382-3390. doi:10.1093/eurheartj/ehx303
